# Supplementary material for: Tuning parameters of dimensionality reduction methods for single-cell RNA-seq analysis
Source: Genome Biol. 2020 Aug 24;21:212. doi: 10.1186/s13059-020-02128-7 (PMC7444048; doi:10.1186/s13059-020-02128-7)
Supplement: Supplementary file 1 — Additional file 1 Contains additional figures and tables for the paper. [file 13059_2020_2128_MOESM1_ESM.pdf]

Additional file 1 for "Tuning parameters of dimensionality reduction methods for single-cell RNA-seq analysis"

Felix Raimundo[1], Celine Vallot[2,3,*], Jean Philippe Vert[1,*]

[1] Google Research, Brain team, 75009 Paris, France
[2] CNRS UMR3244, Institut Curie, PSL Research University, 75005 Paris, France
[3] Translational Research Department, Institut Curie, PSL Research University, 75005 Paris, France

This file contains all supplementary tables and figures mentioned in the main manuscript.

*Correspondance: celine.vallot@curie.fr and jpvert@google.com

Method	Parameter	AMI effect	AMI best	AMI worst	AMI distance
scran	Log normalization	0.504701	logcounts	counts	0.000000
	Dimension of the latent space	0.095888	32	2	0.011168
	Number of top genes	0.046545	300	100	0.070318
	Sum factors normalization	0.000485	0	1	
	ERCC factor normalization	0.000013	0	1	
Seurat	Choice of top features	0.020766	disp	mvp	0.031045
	Number of top genes	0.046823	500	100	0.058046
	Dimension of the latent space	0.090035	10	2	0.006698
	Normalization method	0.139065	LogNormalize	CLR	0.000000
ZinbWave	Dimension of the latent space	0.130836	8.0	128.0	0.127330
	Epsilon	0.034987	2000.0	200.0	0.003970
	Gene covariates	0.005273	1.0	0.0	0.001516
	Number of top genes	0.091435	2000.0	100.0	0.020083
DCA	Dispersion and reconstruction	0.002516	nb	zinb	0.021833
	Batch normalization	0.086200	True	False	0.023997
	Dimension of the latent space	0.202636	32	2	0.017934
	Number of training epochs	0.010789	300	20	0.035311
	Number of hidden neurons	0.010530	64	256	0.026189
	Dropout rate	0.008436	0.1	0	0.053644
	Log normalization	0.035889	True	False	0.001784
	Normalize counts	0.252914	True	False	0.000000
	Scale variance	0.018196	True	False	0.112139
scVI	Dispersion	0.084756	gene	gene-cell	0.000000
	Dropout rate	0.012287	0.1	0	0.005222
	Number of training epochs	0.299515	1000	20	0.052207
	Learning rate	0.180853	0.001	0.0001	0.000000
	Number of hidden neurons	0.008113	128	64	0.067728
	Dimension of the latent space	0.019958	8	64	0.123639
	Layers	0.033922	2	1	0.038795
	Reconstruction loss	0.006086	nb	zinb	0.008399

Table S1: Summary of the parameter influence on the AMI. The column "AMI effect" is the maximum difference between the mean effect of the parameters on the AMI. The column "AMI best" is the parameter value with the best mean effect, and are the ones used in the "ANOVA AMI heuristic". The column "AMI worst" is the parameter value with the worst mean effect. The column "AMI distance" is the maximum distance between the parameter values with the best effect on a dataset specific way, and the "AMI best" effect on a dataset specific way.

Method	Parameter	silhouette effect	silhouette best	silhouette worst	silhouette distance
scran	Log normalization	0.170177	logcounts	counts	0.000000
	Dimension of the latent space	0.167868	8	128	0.132922
	Number of top genes	0.017043	300	3000	0.035144
	Sum factors normalization	0.004166	0	1	
	ERCC factor normalization	0.000000	0	0	
Seurat	Choice of top features	0.006136	disp	mvp	0.026131
	Number of top genes	0.033238	1000	100	0.020503
	Dimension of the latent space	0.250641	2	128	0.119350
	Normalization method	0.080933	LogNormalize	CLR	0.000000
ZinbWave	Dimension of the latent space	0.561425	2.0	128.0	0.000000
	Epsilon	0.031739	2000.0	200.0	0.000000
	Gene covariates	0.000780	1.0	0.0	0.007692
	Number of top genes	0.016251	500.0	100.0	0.059128
DCA	Dispersion and reconstruction	0.017509	nb-conddisp	zinb	0.003257
	Batch normalization	0.032509	True	False	0.043511
	Dimension of the latent space	0.132412	8	128	0.000000
	Number of training epochs	0.018071	20	1000	0.004797
	Number of hidden neurons	0.039340	64	256	0.000000
	Dropout rate	0.001184	0.1	0	0.057457
	Log normalization	0.023018	True	False	0.005908
	Normalize counts	0.117338	True	False	0.000000
	Scale variance	0.005635	True	False	0.055650
scVI	Dispersion	0.070433	gene	gene-cell	0.000000
	Dropout rate	0.018295	0.1	0	0.000000
	Number of training epochs	0.082262	200	20	0.115803
	Learning rate	0.149796	0.001	0.01	0.031726
	Number of hidden neurons	0.028093	64	256	0.017822
	Dimension of the latent space	0.136278	2	32	0.000000
	Layers	0.044539	2	1	0.018577
	Reconstruction loss	0.005252	nb	zinb	0.007876

Table S2: Summary of the parameter influence on the silhouette. The column "silhouette effect" is the maximum difference between the mean effect of the parameters on the silhouette. The column "silhouette best" is the parameter value with the best mean effect, and are the ones used in the "ANOVA silhouette heuristic". The column "silhouette worst" is the parameter value with the worst mean effect. The column "silhouette distance" is the maximum distance between the parameter values with the best effect on a dataset specific way, and the "silhouette best" effect on a dataset specific way.

	SS	df	MS	F value	Pr(>F)
scran_n_tops	0.45	5.00	0.09	35.11	0.0000
dataset	22.93	9.00	2.55	982.90	0.0000
scran_sum_factor	0.00	1.00	0.00	0.05	0.8268
scran_ercc	0.00	1.00	0.00	0.00	0.9968
scran_assay	135.85	1.00	135.85	52420.33	0.0000
scran_n_pcs	2.06	7.00	0.29	113.30	0.0000
scran_n_tops:dataset	3.74	45.00	0.08	32.04	0.0000
dataset:scran_assay	21.32	9.00	2.37	913.92	0.0000
dataset:scran_n_pcs	0.60	63.00	0.01	3.70	0.0000
Residuals	5.60	2162.00	0.00		

Table S3: Summary result of the ANOVA for the influence of the parameters of scran on its AMI. "SS" corresponds to the variance explained by a parameter, "df" its number of degrees of freedom, "MS" is "SS" divided by "df", i.e. the mean variance explained by each degree of freedom, "F value", is the observed F statistic, and "Pr(>F)" is the probability under the null hypothesis (this parameter has no influence on the AMI) to observe an F statistic this high. Each row corresponds to a factor, when they follow the format "parameter" it is the effect that this parameter has on average, when they follow the format "dataset:parameter" it is the effect of the parameter on each specific dataset (the interaction factors), "dataset" is a special one that represents the effect of the dataset on the AMI, it is used to represent the inherent complexity of the data.

	SS	df	MS	F value	Pr(>F)
dataset	19.50	9.00	2.17	591.87	0.0000
scran_n_tops	0.07	5.00	0.01	4.06	0.0011
scran_sum_factor	0.01	1.00	0.01	2.50	0.1138
scran_ercc	0.00	1.00	0.00	0.00	0.9974
scran_assay	15.45	1.00	15.45	4218.86	0.0000
scran_n_pcs	6.94	7.00	0.99	270.77	0.0000
dataset:scran_n_tops	0.42	45.00	0.01	2.57	0.0000
dataset:scran_assay	3.70	9.00	0.41	112.44	0.0000
dataset:scran_n_pcs	2.25	63.00	0.04	9.75	0.0000
Residuals	7.92	2162.00	0.00		

Table S4: Summary result of the ANOVA for the influence of the parameters of scran on its silhouette. "SS" corresponds to the variance explained by a parameter, "df" its number of degrees of freedom, "MS" is "SS" divided by "df", i.e. the mean variance explained by each degree of freedom, "F value", is the observed F statistic, and "Pr(>F)" is the probability under the null hypothesis (this parameter has no influence on the silhouette) to observe an F statistic this high.

Each row corresponds to a factor, when they follow the format "parameter" it is the effect that this parameter has on average, when they follow the format "dataset:parameter" it is the effect of the parameter on each specific dataset (the interaction factors), "dataset" is a special one that represents the effect of the dataset on the silhouette, it is used to represent the inherent complexity of the data.

	SS	df	MS	F value	Pr(>F)
dataset	103.26	9.00	11.47	2711.13	0.0000
seurat_n_features	0.75	5.00	0.15	35.53	0.0000
seurat_n_pcs	2.49	7.00	0.36	84.12	0.0000
seurat_norm	13.92	1.00	13.92	3289.15	0.0000
seurat_find_variable	0.23	2.00	0.12	27.52	0.0000
dataset:seurat_n_features	2.51	45.00	0.06	13.20	0.0000
dataset:seurat_n_pcs	1.12	63.00	0.02	4.20	0.0000
dataset:seurat_norm	11.95	9.00	1.33	313.81	0.0000
dataset:seurat_find_variable	1.05	18.00	0.06	13.84	0.0000
Residuals	11.51	2719.00	0.00		

Table S5: Summary result of the ANOVA for the influence of the parameters of Seurat on its AMI. "SS" corresponds to the variance explained by a parameter, "df" its number of degrees of freedom, "MS" is "SS" divided by "df", i.e. the mean variance explained by each degree of freedom, "F value", is the observed F statistic, and "Pr(>F)" is the probability under the null hypothesis (this parameter has no influence on the AMI) to observe an F statistic this high. Each row corresponds to a factor, when they follow the format "parameter" it is the effect that this parameter has on average, when they follow the format "dataset:parameter" it is the effect of the parameter on each specific dataset (the interaction factors), "dataset" is a special one that represents the effect of the dataset on the AMI, it is used to represent the inherent complexity of the data.

	SS	df	MS	F value	Pr(>F)
dataset	62.00	9.00	6.89	2855.27	0.0000
seurat_n_features	0.36	5.00	0.07	30.15	0.0000
seurat_n_pcs	21.87	7.00	3.12	1295.01	0.0000
seurat_norm	4.71	1.00	4.71	1953.86	0.0000
seurat_find_variable	0.02	2.00	0.01	3.92	0.0200
dataset:seurat_n_features	0.51	45.00	0.01	4.66	0.0000
dataset:seurat_n_pcs	7.03	63.00	0.11	46.28	0.0000
dataset:seurat_norm	0.57	9.00	0.06	26.12	0.0000
dataset:seurat_find_variable	0.42	18.00	0.02	9.56	0.0000
Residuals	6.56	2719.00	0.00		

Table S6: Summary result of the ANOVA for the influence of the parameters of Seurat on its silhouette. "SS" corresponds to the variance explained by a parameter, "df" its number of degrees of freedom, "MS" is "SS" divided by "df", i.e. the mean variance explained by each degree of freedom, "F value", is the observed F statistic, and "Pr(>F)" is the probability under the null hypothesis (this parameter has no influence on the silhouette) to observe an F statistic this high.
Each row corresponds to a factor, when they follow the format "parameter" it is the effect that this parameter has on average, when they follow the format "dataset:parameter" it is the effect of the parameter on each specific dataset (the interaction factors), "dataset" is a special one that represents the effect of the dataset on the silhouette, it is used to represent the inherent complexity of the data.

	SS	df	MS	F value	Pr(>F)
dataset	186.02	9.00	20.67	6293.87	0.0000
zinbwave_dims	5.10	7.00	0.73	221.82	0.0000
zinbwave_epsilon	0.51	3.00	0.17	51.78	0.0000
zinbwave_gene_covariate	0.02	1.00	0.02	6.49	0.0109
zinbwave_keep_variance	2.65	4.00	0.66	201.45	0.0000
dataset:zinbwave_dims	12.67	63.00	0.20	61.22	0.0000
dataset:zinbwave_epsilon	0.81	27.00	0.03	9.14	0.0000
dataset:zinbwave_gene_covariate	0.03	9.00	0.00	0.93	0.5013
dataset:zinbwave_keep_variance	2.18	36.00	0.06	18.45	0.0000
Residuals	9.56	2910.00	0.00		

Table S7: Summary result of the ANOVA for the influence of the parameters of ZinbWave on its AMI. "SS" corresponds to the variance explained by a parameter, "df" its number of degrees of freedom, "MS" is "SS" divided by "df", i.e. the mean variance explained by each degree of freedom, "F value", is the observed F statistic, and "Pr(>F)" is the probability under the null hypothesis (this parameter has no influence on the AMI) to observe an F statistic this high.
Each row corresponds to a factor, when they follow the format "parameter" it is the effect that this parameter has on average, when they follow the format "dataset:parameter" it is the effect of the parameter on each specific dataset (the interaction factors), "dataset" is a special one that represents the effect of the dataset on the AMI, it is used to represent the inherent complexity of the data.

	SS	df	MS	F value	Pr(>F)
dataset	22.65	9.00	2.52	2978.44	0.0000
zinbwave_dims	96.94	7.00	13.85	16391.25	0.0000
zinbwave_epsilon	0.43	3.00	0.14	168.12	0.0000
zinbwave_gene_covariate	0.00	1.00	0.00	0.55	0.4577
zinbwave_keep_variance	0.10	4.00	0.03	30.21	0.0000
dataset:zinbwave_dims	17.16	63.00	0.27	322.33	0.0000
dataset:zinbwave_epsilon	0.08	27.00	0.00	3.65	0.0000
dataset:zinbwave_gene_covariate	0.04	9.00	0.00	4.82	0.0000
dataset:zinbwave_keep_variance	0.98	36.00	0.03	32.08	0.0000
Residuals	2.46	2910.00	0.00		

Table S8: Summary result of the ANOVA for the influence of the parameters of ZinbWave on its silhouette. "SS" corresponds to the variance explained by a parameter, "df" its number of degrees of freedom, "MS" is "SS" divided by "df", i.e. the mean variance explained by each degree of freedom, "F value", is the observed F statistic, and "Pr(>F)" is the probability under the null hypothesis (this parameter has no influence on the silhouette) to observe an F statistic this high.
Each row corresponds to a factor, when they follow the format "parameter" it is the effect that this parameter has on average, when they follow the format "dataset:parameter" it is the effect of the parameter on each specific dataset (the interaction factors), "dataset" is a special one that represents the effect of the dataset on the silhouette, it is used to represent the inherent complexity of the data.

	SS	df	MS	F value	Pr(>F)
dataset	6045.41	9.00	671.71	50549.34	0.0000
ae_type	0.21	3.00	0.07	5.28	0.0012
batchnorm	381.96	1.00	381.96	28744.52	0.0000
dims	864.32	7.00	123.47	9292.01	0.0000
epochs	2.66	6.00	0.44	33.39	0.0000
normalize_per_cell	3268.93	1.00	3268.93	246001.82	0.0000
scale	16.85	1.00	16.85	1268.15	0.0000
log1p	64.31	1.00	64.31	4839.39	0.0000
hidden_dropout	3.47	1.00	3.47	261.21	0.0000
hidden	3.95	2.00	1.97	148.48	0.0000
dataset:ae_type	15.73	27.00	0.58	43.84	0.0000
dataset:batchnorm	731.66	9.00	81.30	6117.89	0.0000
dataset:dims	135.71	63.00	2.15	162.11	0.0000
dataset:epochs	14.74	54.00	0.27	20.54	0.0000
dataset:normalize_per_cell	1432.67	9.00	159.19	11979.42	0.0000
dataset:scale	294.29	9.00	32.70	2460.72	0.0000
dataset:log1p	145.38	9.00	16.15	1215.58	0.0000
dataset:hidden_dropout	58.16	9.00	6.46	486.27	0.0000
dataset:hidden	29.01	18.00	1.61	121.30	0.0000
Residuals	2727.97	205292.00	0.01		

Table S9: Summary result of the ANOVA for the influence of the parameters of DCA on its AMI. "SS" corresponds to the variance explained by a parameter, "df" its number of degrees of freedom, "MS" is "SS" divided by "df", i.e. the mean variance explained by each degree of freedom, "F value", is the observed F statistic, and "Pr(>F)" is the probability under the null hypothesis (this parameter has no influence on the AMI) to observe an F statistic this high. Each row corresponds to a factor, when they follow the format "parameter" it is the effect that this parameter has on average, when they follow the format "dataset:parameter" it is the effect of the parameter on each specific dataset (the interaction factors), "dataset" is a special one that represents the effect of the dataset on the AMI, it is used to represent the inherent complexity of the data.

	SS	df	MS	F value	Pr(>F)
dataset	3542.22	9.00	393.58	46229.81	0.0000
ae_type	9.36	3.00	3.12	366.43	0.0000
batchnorm	53.66	1.00	53.66	6303.34	0.0000
dims	396.37	7.00	56.62	6651.01	0.0000
epochs	7.71	6.00	1.28	150.92	0.0000
normalize_per_cell	700.98	1.00	700.98	82336.34	0.0000
scale	1.56	1.00	1.56	183.13	0.0000
log1p	26.88	1.00	26.88	3157.50	0.0000
hidden_dropout	0.06	1.00	0.06	7.41	0.0065
hidden	52.00	2.00	26.00	3054.02	0.0000
dataset:ae_type	7.71	27.00	0.29	33.56	0.0000
dataset:batchnorm	494.51	9.00	54.95	6453.93	0.0000
dataset:dims	124.46	63.00	1.98	232.05	0.0000
dataset:epochs	8.94	54.00	0.17	19.46	0.0000
dataset:normalize_per_cell	131.01	9.00	14.56	1709.83	0.0000
dataset:scale	142.09	9.00	15.79	1854.47	0.0000
dataset:log1p	72.51	9.00	8.06	946.32	0.0000
dataset:hidden_dropout	37.63	9.00	4.18	491.17	0.0000
dataset:hidden	14.03	18.00	0.78	91.53	0.0000
Residuals	1747.77	205292.00	0.01		

Table S10: Summary result of the ANOVA for the influence of the parameters of DCA on its silhouette. "SS" corresponds to the variance explained by a parameter, "df" its number of degrees of freedom, "MS" is "SS" divided by "df", i.e. the mean variance explained by each degree of freedom, "F value", is the observed F statistic, and "Pr(>F)" is the probability under the null hypothesis (this parameter has no influence on the silhouette) to observe an F statistic this high.
Each row corresponds to a factor, when they follow the format "parameter" it is the effect that this parameter has on average, when they follow the format "dataset:parameter" it is the effect of the parameter on each specific dataset (the interaction factors), "dataset" is a special one that represents the effect of the dataset on the silhouette, it is used to represent the inherent complexity of the data.

	SS	df	MS	F value	Pr(>F)
n_latent	4.07	7.00	0.58	12.56	0.0000
dataset	3304.57	9.00	367.17	7934.53	0.0000
epochs	836.88	6.00	139.48	3014.13	0.0000
dispersion	143.17	1.00	143.17	3093.80	0.0000
n_layers	23.07	1.00	23.07	498.51	0.0000
n_hidden	0.90	2.00	0.45	9.78	0.0001
dropout_rate	2.98	1.00	2.98	64.44	0.0000
lr	471.85	2.00	235.92	5098.25	0.0000
reconstruction_loss	0.76	1.00	0.76	16.35	0.0001
n_latent:dataset	39.76	63.00	0.63	13.64	0.0000
dataset:epochs	383.74	54.00	7.11	153.57	0.0000
dataset:dispersion	240.35	9.00	26.71	577.09	0.0000
dataset:n_layers	54.17	9.00	6.02	130.08	0.0000
dataset:n_hidden	61.33	18.00	3.41	73.63	0.0000
dataset:dropout_rate	5.08	9.00	0.56	12.20	0.0000
dataset:lr	657.45	18.00	36.53	789.30	0.0000
dataset:reconstruction_loss	2.88	9.00	0.32	6.91	0.0000
Residuals	3679.78	79519.00	0.05		

Table S11: Summary result of the ANOVA for the influence of the parameters of scVI on its AMI. "SS" corresponds to the variance explained by a parameter, "df" its number of degrees of freedom, "MS" is "SS" divided by "df", i.e. the mean variance explained by each degree of freedom, "F value", is the observed F statistic, and "Pr(>F)" is the probability under the null hypothesis (this parameter has no influence on the AMI) to observe an F statistic this high. Each row corresponds to a factor, when they follow the format "parameter" it is the effect that this parameter has on average, when they follow the format "dataset:parameter" it is the effect of the parameter on each specific dataset (the interaction factors), "dataset" is a special one that represents the effect of the dataset on the AMI, it is used to represent the inherent complexity of the data.

	SS	df	MS	F value	Pr(>F)
n_latent	151.33	7.00	21.62	618.87	0.0000
dataset	914.48	9.00	101.61	2908.80	0.0000
epochs	61.21	6.00	10.20	292.04	0.0000
dispersion	98.93	1.00	98.93	2832.20	0.0000
n_layers	39.34	1.00	39.34	1126.30	0.0000
n_hidden	10.57	2.00	5.28	151.29	0.0000
dropout_rate	6.65	1.00	6.65	190.50	0.0000
lr	302.15	2.00	151.07	4324.90	0.0000
reconstruction_loss	0.53	1.00	0.53	15.27	0.0001
n_latent:dataset	56.63	63.00	0.90	25.73	0.0000
dataset:epochs	293.43	54.00	5.43	155.56	0.0000
dataset:dispersion	130.38	9.00	14.49	414.73	0.0000
dataset:n_layers	27.01	9.00	3.00	85.91	0.0000
dataset:n_hidden	32.82	18.00	1.82	52.19	0.0000
dataset:dropout_rate	1.94	9.00	0.22	6.18	0.0000
dataset:lr	410.50	18.00	22.81	652.87	0.0000
dataset:reconstruction_loss	0.91	9.00	0.10	2.88	0.0021
Residuals	2777.70	79519.00	0.03		

Table S12: Summary result of the ANOVA for the influence of the parameters of scVI on its silhouette. "SS" corresponds to the variance explained by a parameter, "df" its number of degrees of freedom, "MS" is "SS" divided by "df", i.e. the mean variance explained by each degree of freedom, "F value", is the observed F statistic, and "Pr(>F)" is the probability under the null hypothesis (this parameter has no influence on the silhouette) to observe an F statistic this high.
Each row corresponds to a factor, when they follow the format "parameter" it is the effect that this parameter has on average, when they follow the format "dataset:parameter" it is the effect of the parameter on each specific dataset (the interaction factors), "dataset" is a special one that represents the effect of the dataset on the silhouette, it is used to represent the inherent complexity of the data.

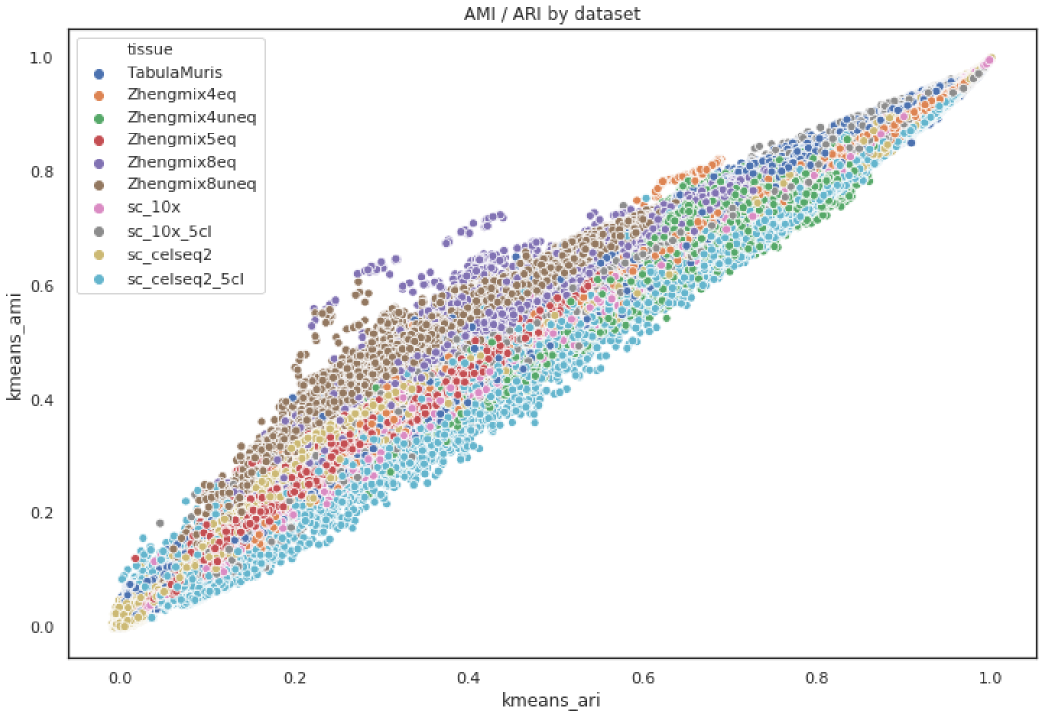

Figure S1: Relationship between AMI (vertical axis) and ARI (horizontal axis), computed with k-means clustering, colored by dataset. Each point represents the result of one experiment (running one method with a particular set of parameters on one dataset). We see that, overall, ARI and AMI are strongly correlated, particularly for a given dataset.

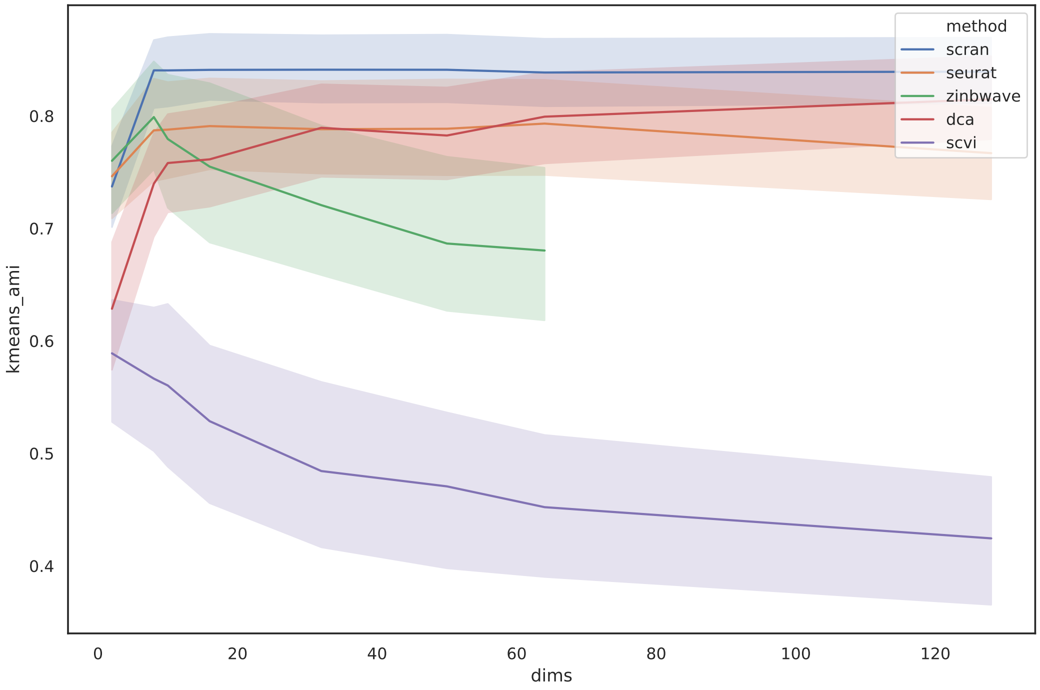

Figure S2: Mean AMI score across the 10 datasets (solid lines) for each method with default parameters. The transparent lines are the 95% confidence interval, which is large since we only have samples per point. The x axis is the dimension of the latent space, in order to observe its effect on the AMI.

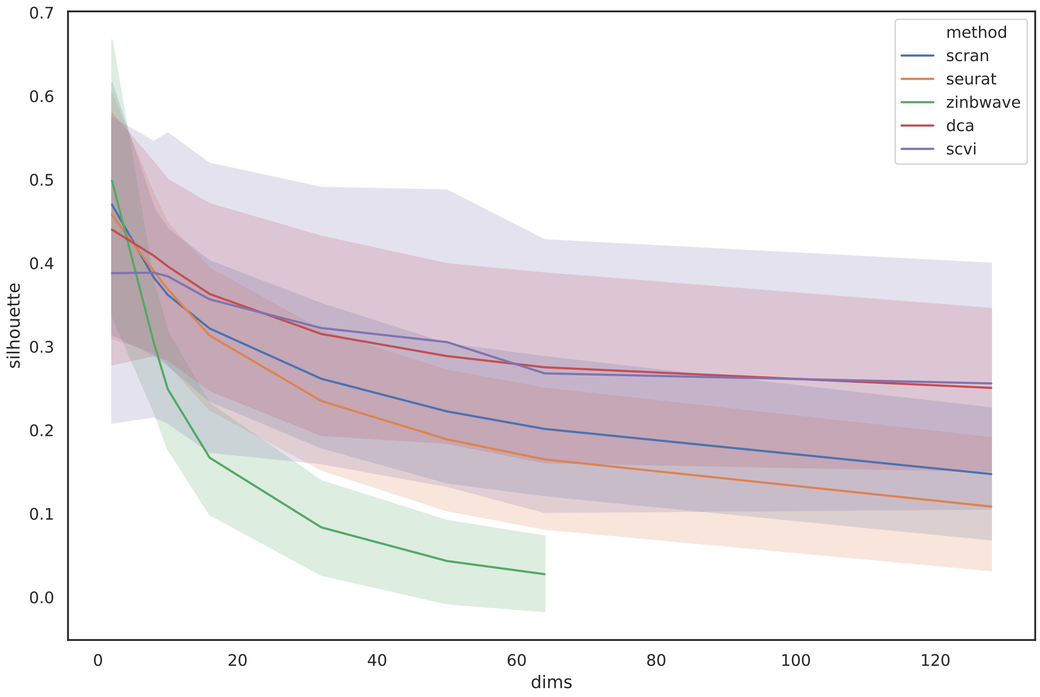

Figure S3: Mean silhouette score across the 10 datasets (solid lines) for each method with default parameters. The transparent lines are the 95% confidence interval, which is large since we only have samples per point. The x axis is the dimension of the latent space, in order to observe its effect on the silhouette.

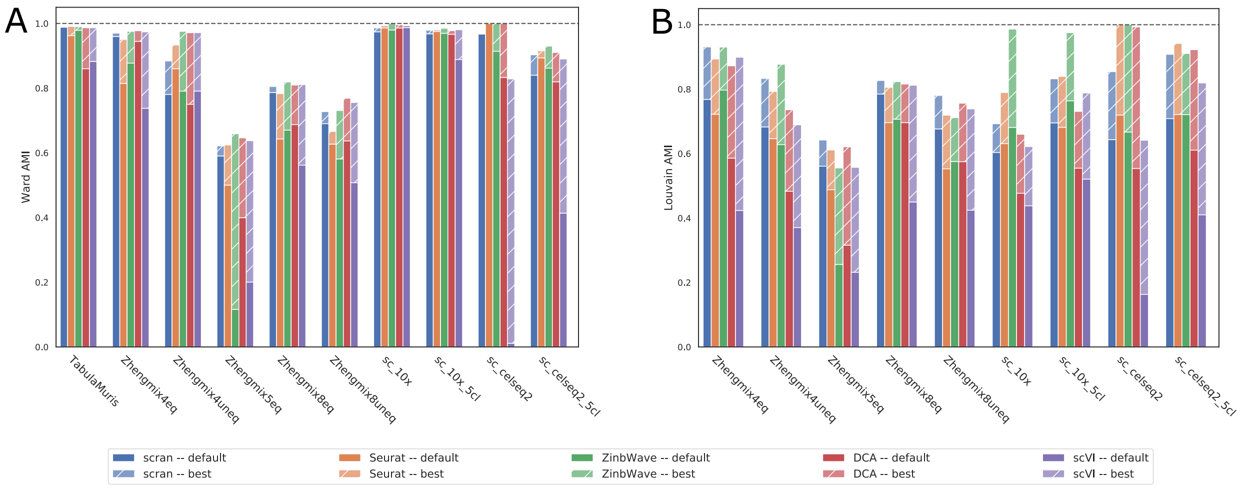

Figure S4: AMI after **A.** Ward clustering and **B.** Louvain clustering (right) of five DR pipelines (scran, Seurat, ZinbWave, DCA and scVI) with default parameters and a dimension of 10 (legend "default") or after parameter optimization (legend "best") on our benchmark of ten datasets.

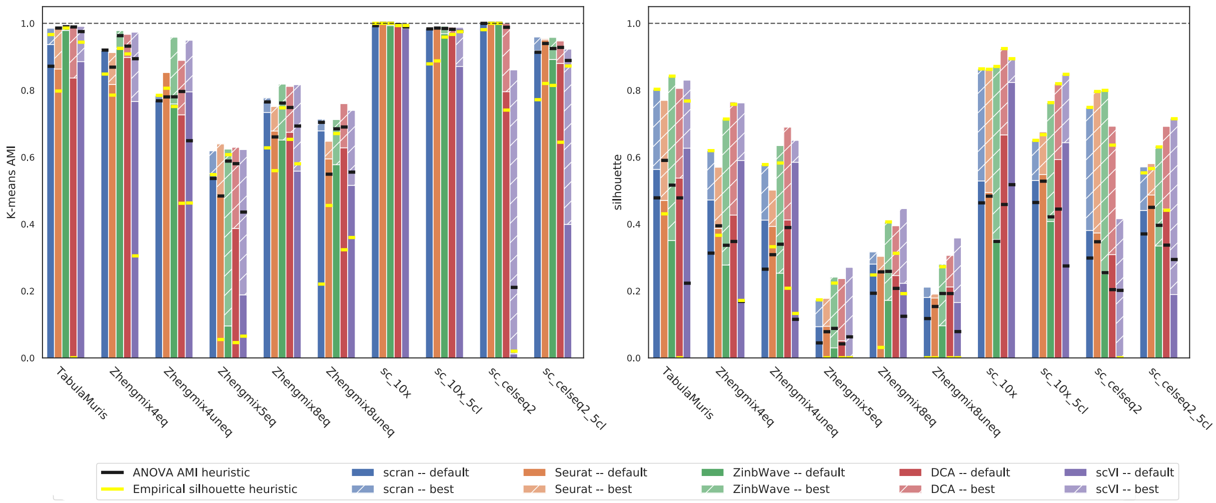

Figure S5: Performance of five DR pipelines (scran, Seurat, ZinbWave, DCA and scVI) with default parameters and a dimension of 10 (legend "default") or after parameter optimization (legend "best") on our benchmark of ten dataset. The "ANOVA AMI heuristic" corresponds to the performances of the new default parameters. The "Empirical silhouette heuristic" corresponds to the performance of the heuristic using the best empirical silhouette.

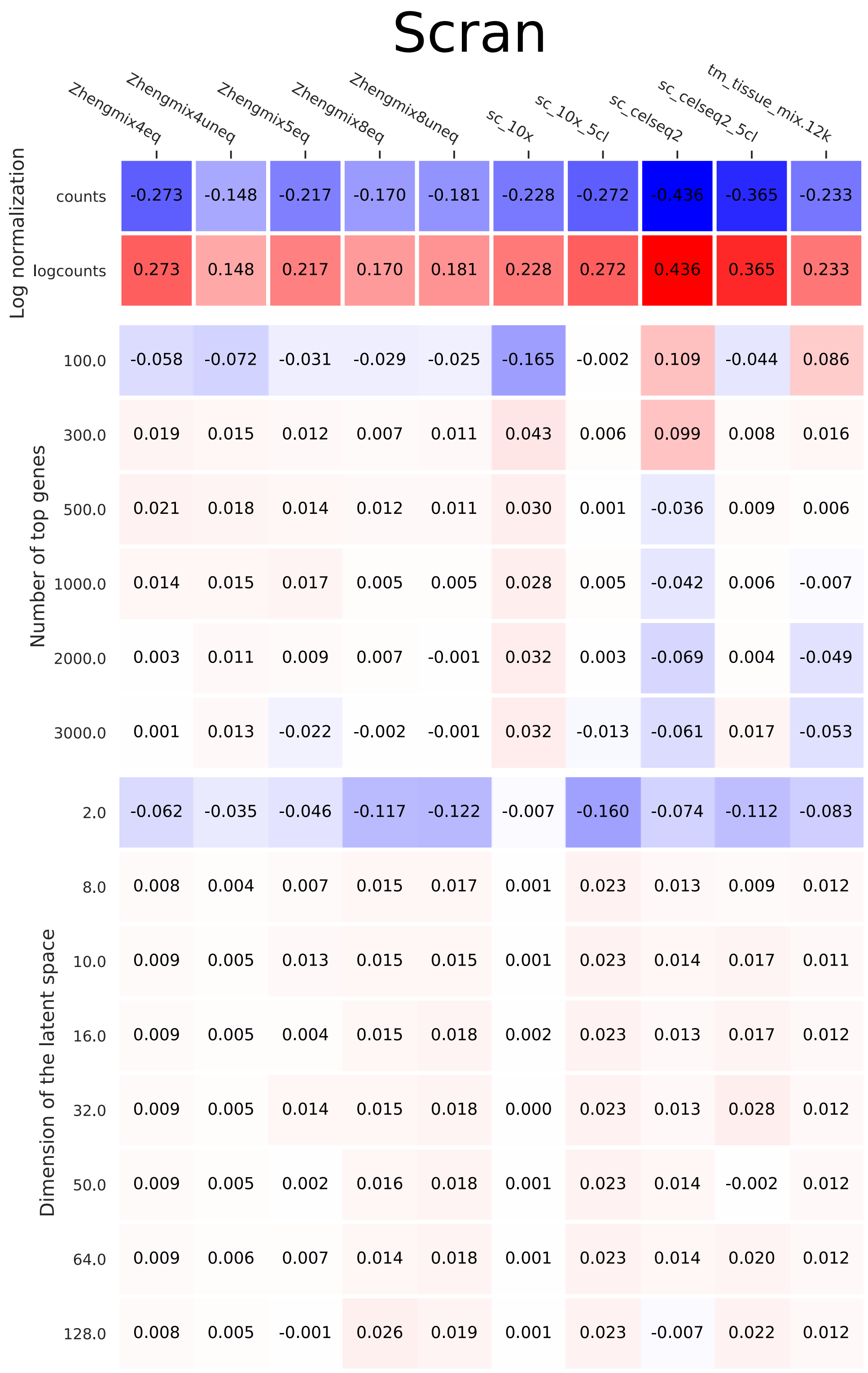

Figure S6: Heatmap visualization of the mean effect of each parameter value of scran on its AMI. Each columns corresponds to a dataset. The rows are split by parameter and their values, the numbers show the average effect of that parameter value on the AMI compared to the mean AMI for scran on that dataset.
These effects come from a factorial ANOVA.

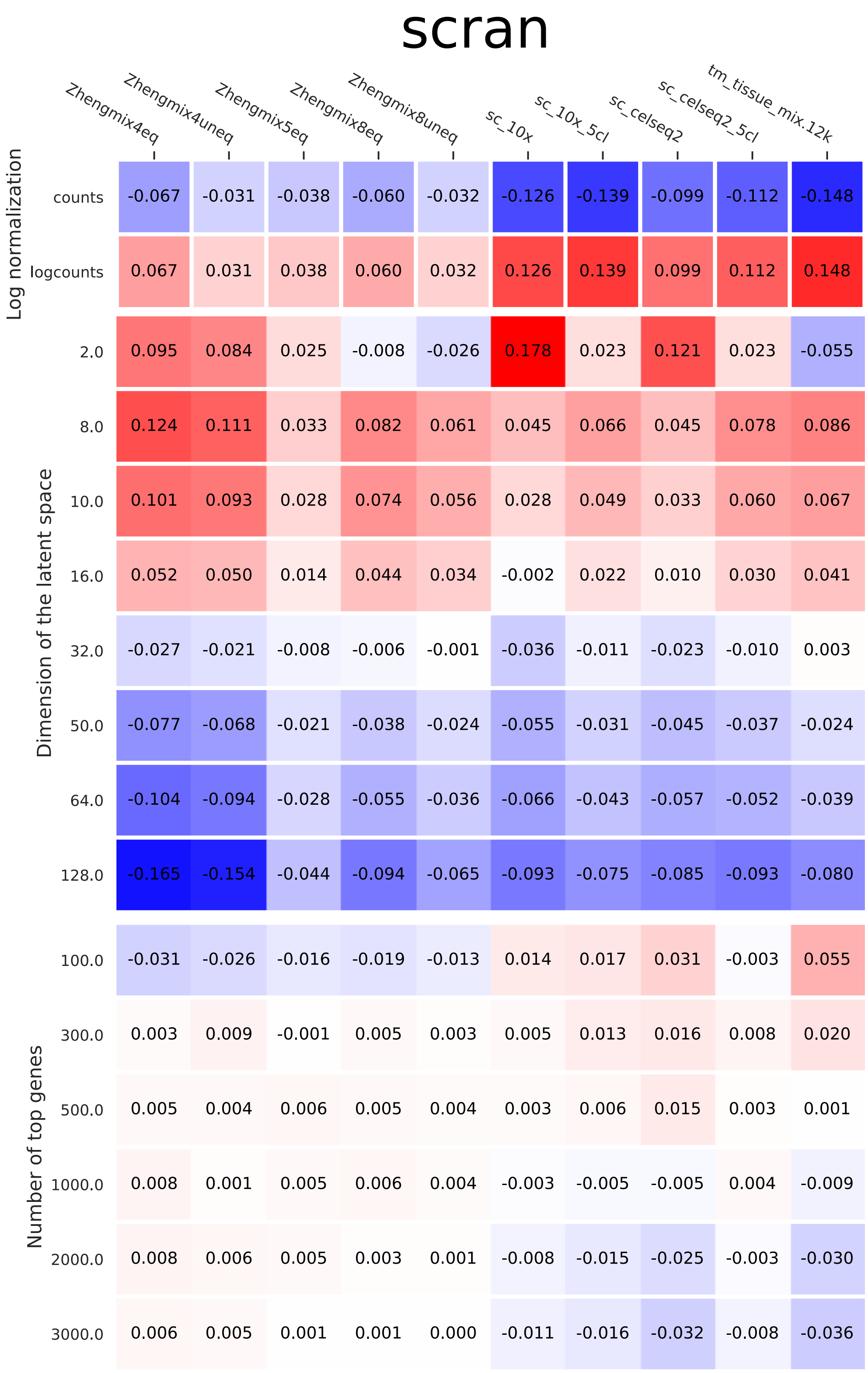

Figure S7: Heatmap visualization of the mean effect of each parameter value of scran on its silhouette. Each columns corresponds to a dataset. The rows are split by parameter and their values, the numbers show the average effect of that parameter value on the silhouette compared to the mean silhouette for scran on that dataset.
These effects come from a factorial ANOVA.

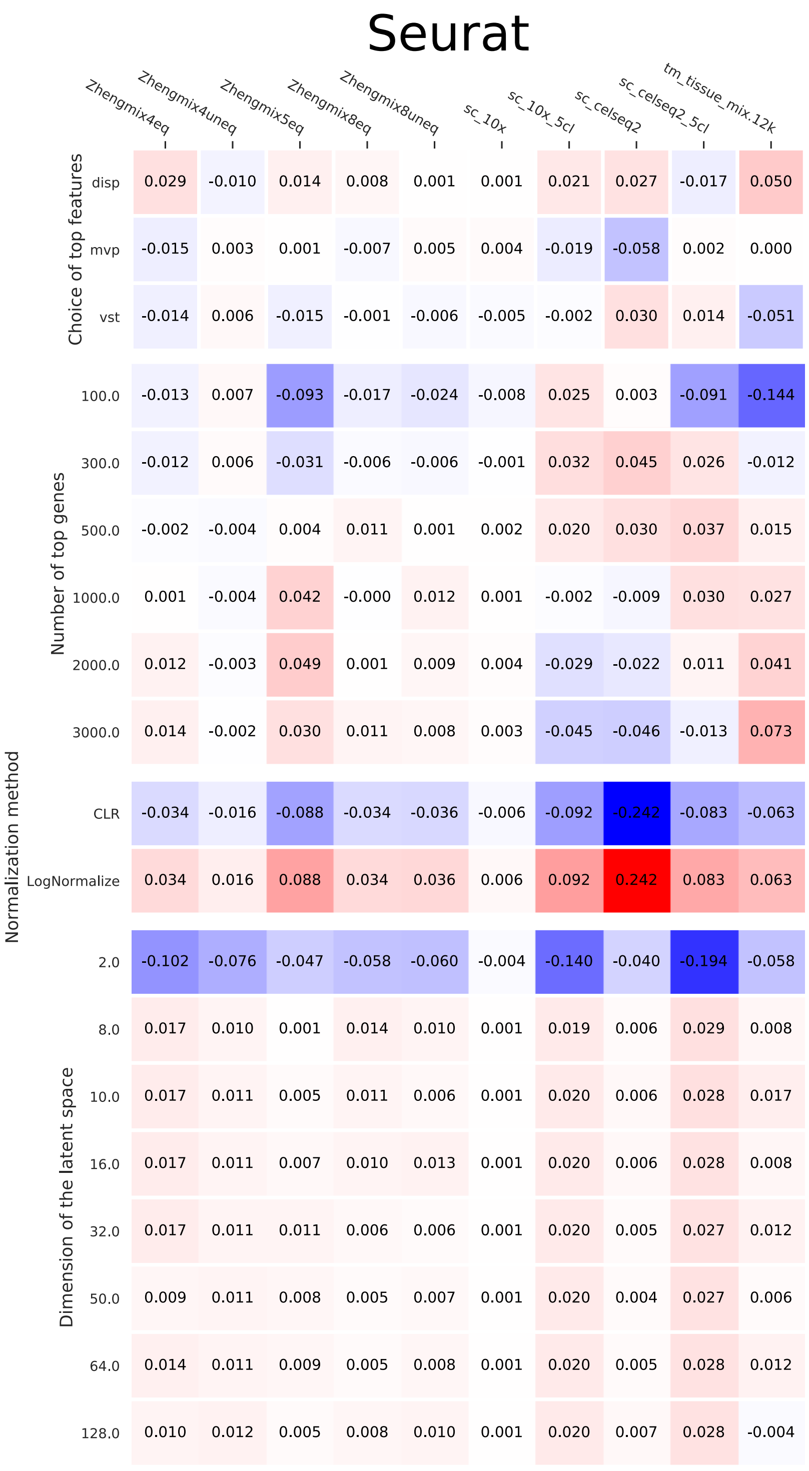

Figure S8: Heatmap visualization of the mean effect of each parameter value of Seurat on its AMI. Each columns corresponds to a dataset. The rows are split by parameter and their values, the numbers show the average effect of that parameter value on the AMI compared to the mean AMI for Seurat on that dataset.
These effects come from a factorial ANOVA.

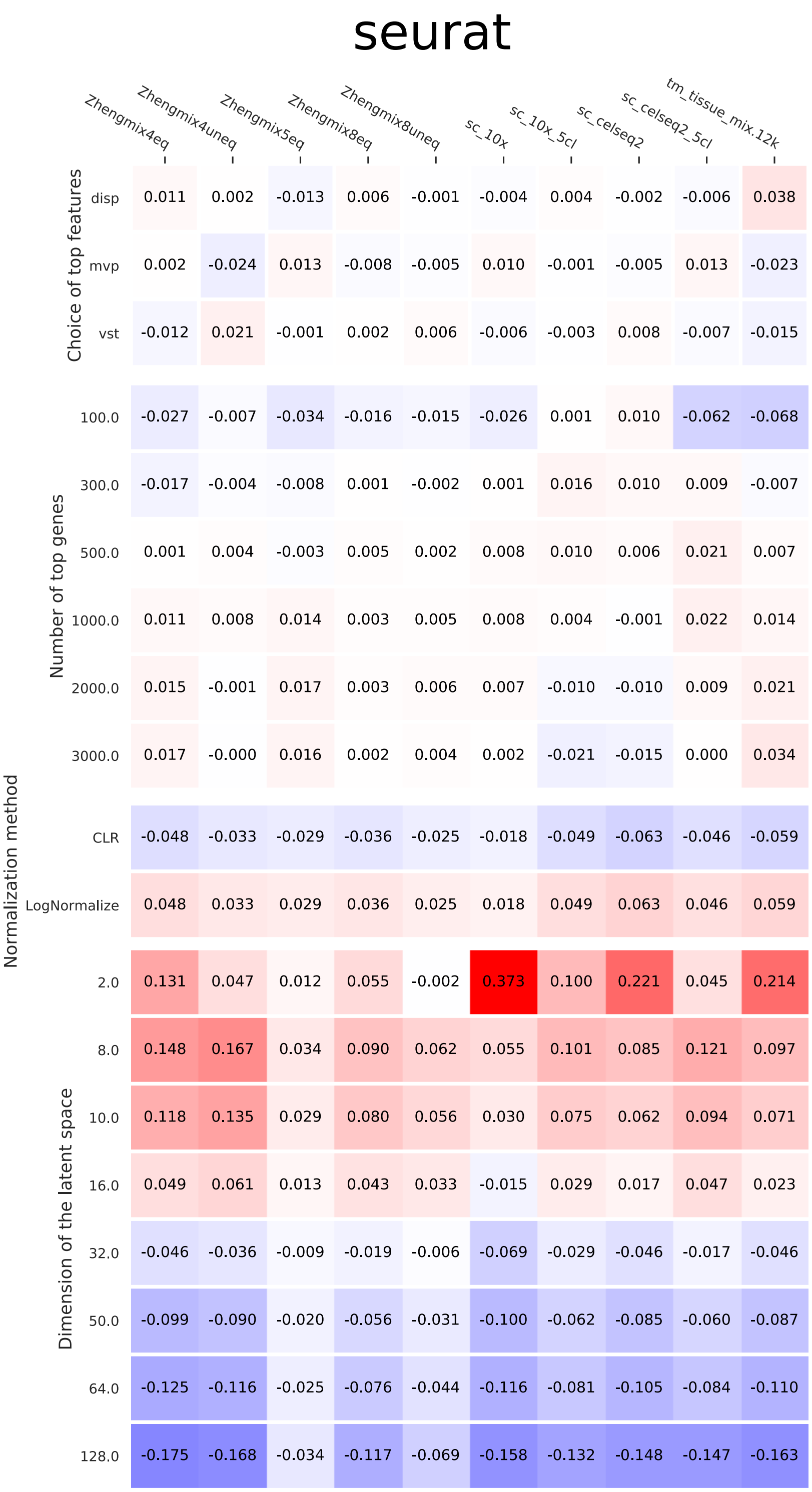

Figure S9: Heatmap visualization of the mean effect of each parameter value of Seurat on its silhouette. Each columns corresponds to a dataset. The rows are split by parameter and their values, the numbers show the average effect of that parameter value on the silhouette compared to the mean silhouette for Seurat on that dataset.
These effects come from a factorial ANOVA.

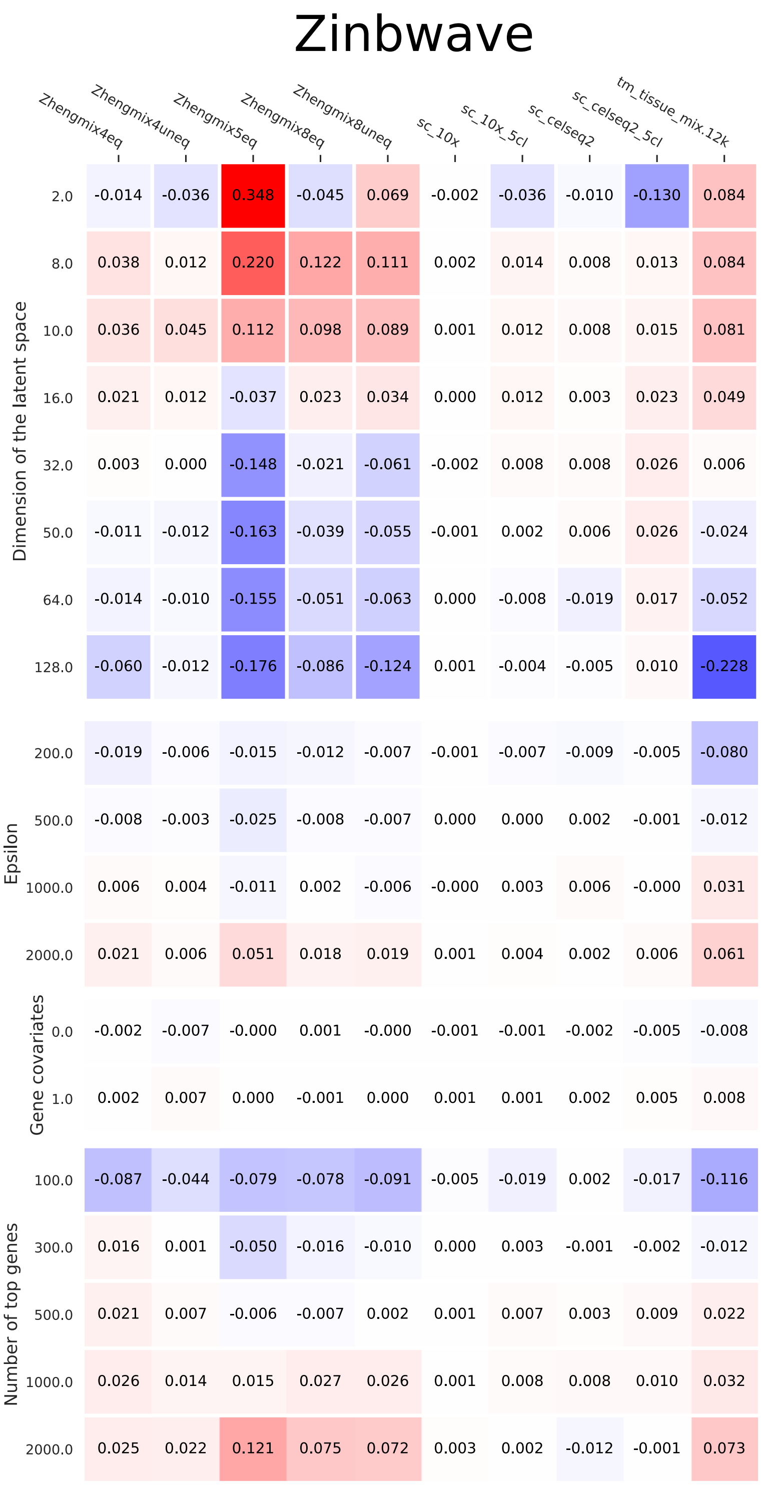

Figure S10: Heatmap visualization of the mean effect of each parameter value of ZinbWave on its AMI. Each columns corresponds to a dataset. The rows are split by parameter and their values, the numbers show the average effect of that parameter value on the AMI compared to the mean AMI for ZinbWave on that dataset.
These effects come from a factorial ANOVA.

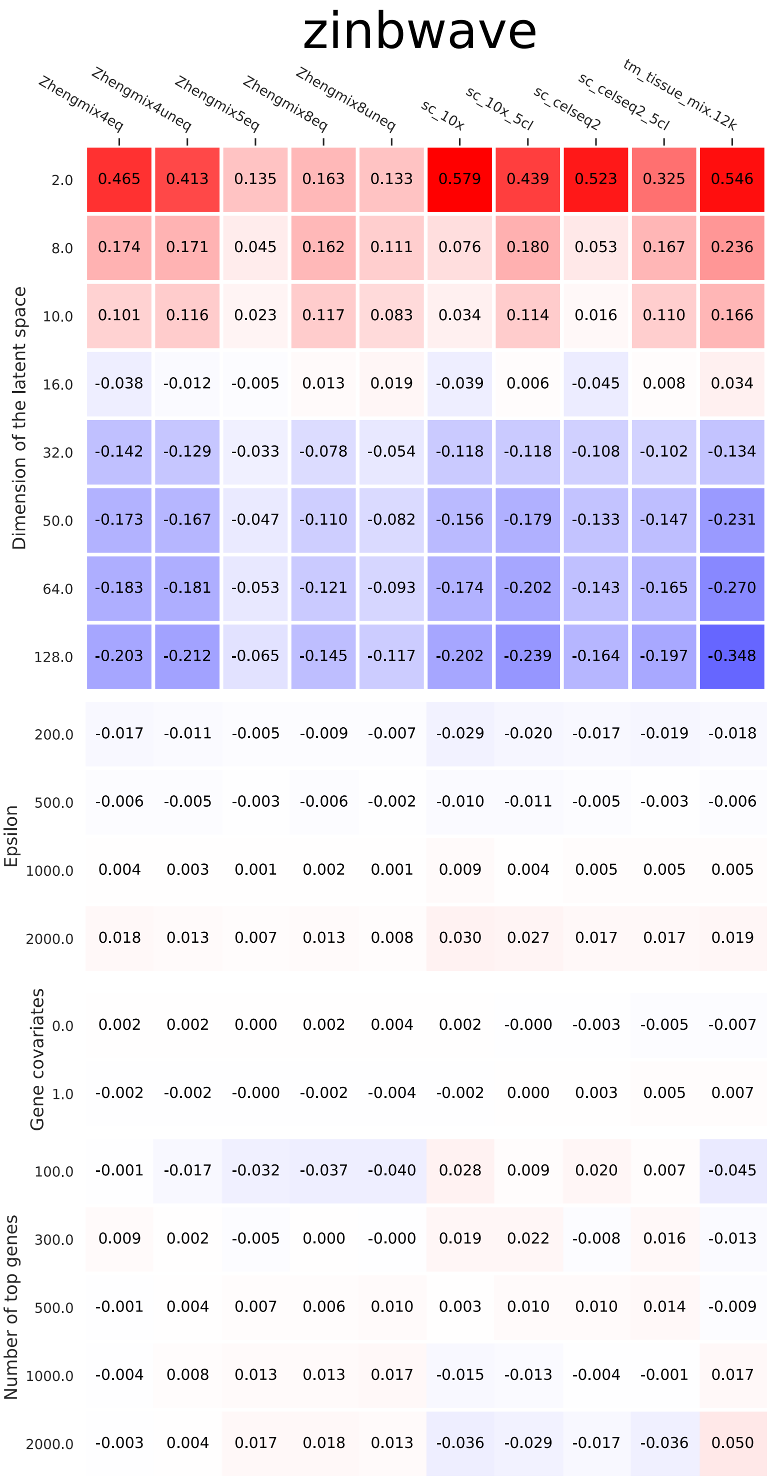

Figure S11: Heatmap visualization of the mean effect of each parameter value of ZinbWave on its silhouette Each columns corresponds to a dataset. The rows are split by parameter and their values, the numbers show the average effect of that parameter value on the silhouette compared to the mean silhouette for ZinbWave on that dataset.
These effects come from a factorial ANOVA.

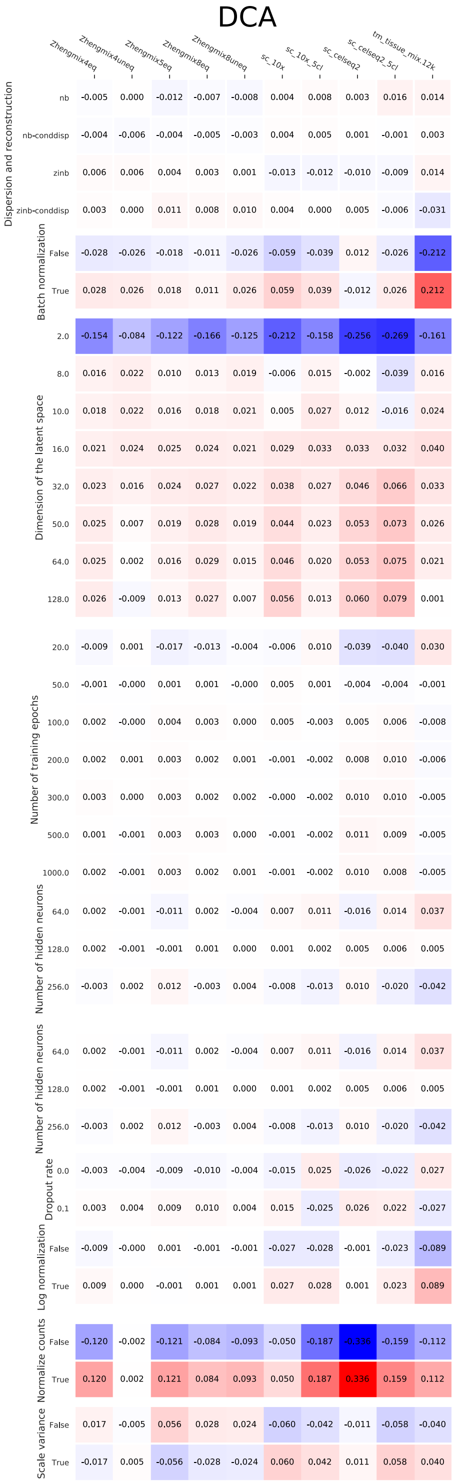

Figure S12: Heatmap visualization of the mean effect of each parameter value of DCA on its AMI. Each columns corresponds to a dataset. The rows are split by parameter and their values, the numbers show the average effect of that parameter value on the AMI compared to the mean AMI for DCA on that dataset.
These effects come from a factorial ANOVA.

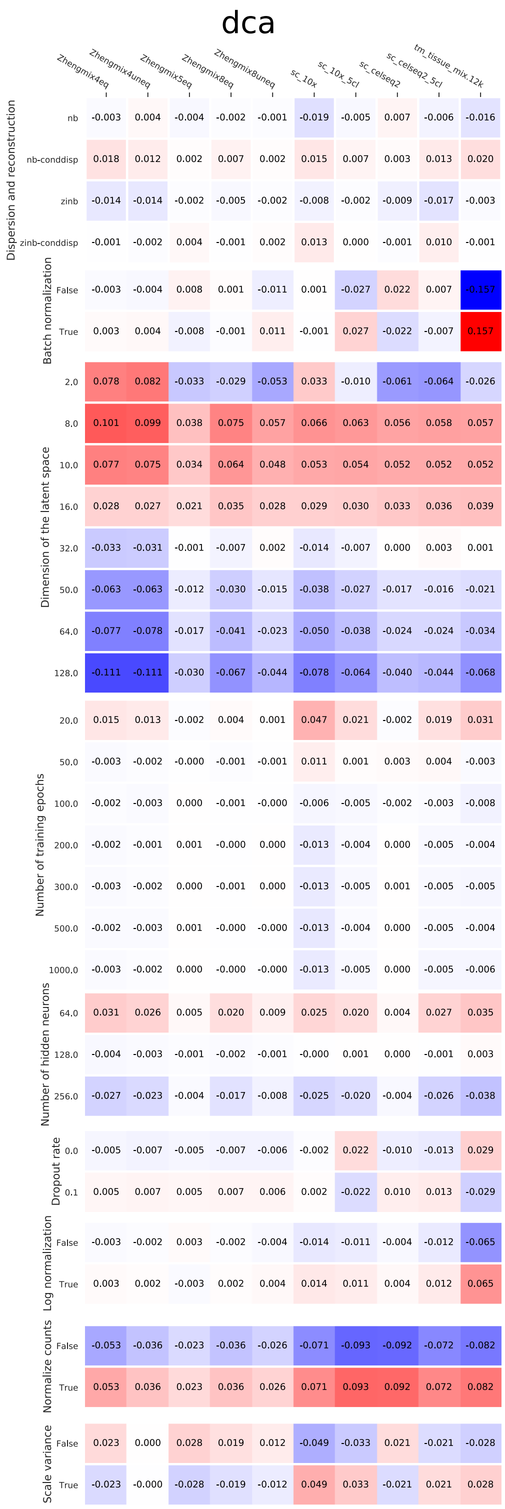

Figure S13: Heatmap visualization of the mean effect of each parameter value of DCA on its silhouette. Each columns corresponds to a dataset. The rows are split by parameter and their values, the numbers show the average effect of that parameter value on the silhouette compared to the mean silhouette for DCA on that dataset.
These effects come from a factorial ANOVA.

scVI

Parameter	Value	Zhengmix4eq	Zhengmix4uneq	Zhengmix5eq	Zhengmix8eq	Zhengmix8uneq	sc_10x	sc_10x_5cl	sc_celseq2	sc_celseq2_5cl	tm_tissue_mix.12k
Dispersion	gene	0.018	0.007	0.005	0.006	0.006	0.164	0.050	0.019	0.016	0.132
	gene-cell	-0.018	-0.007	-0.005	-0.006	-0.006	-0.164	-0.050	-0.019	-0.016	-0.132
Dropout rate	0.0	-0.005	0.000	0.002	0.002	-0.002	-0.004	-0.007	-0.009	-0.024	-0.016
	0.1	0.005	-0.000	-0.002	-0.002	0.002	0.004	0.007	0.009	0.024	0.016
Number of training epochs	20.0	-0.268	-0.072	-0.169	-0.220	-0.139	-0.200	-0.388	-0.056	-0.290	-0.112
	50.0	-0.091	-0.017	-0.055	-0.067	-0.036	-0.175	-0.173	-0.055	-0.247	-0.039
	100.0	-0.013	0.027	-0.002	-0.008	0.016	-0.099	-0.038	-0.038	-0.124	0.016
	200.0	0.060	0.031	0.035	0.050	0.050	0.014	0.091	-0.021	0.041	0.058
	300.0	0.101	0.027	0.057	0.080	0.055	0.076	0.143	0.002	0.109	0.045
	500.0	0.117	0.017	0.075	0.090	0.044	0.151	0.180	0.051	0.198	0.026
	1000.0	0.093	-0.013	0.060	0.074	0.010	0.233	0.185	0.117	0.313	0.006
Learning rate	0.0001	-0.145	-0.045	-0.052	-0.111	-0.062	-0.158	-0.106	-0.033	-0.182	0.147
	0.001	0.092	0.031	0.047	0.067	0.038	0.213	0.146	0.052	0.171	0.201
	0.01	0.054	0.014	0.005	0.045	0.024	-0.055	-0.040	-0.019	0.011	-0.348
Number of hidden neurons	64.0	-0.033	-0.018	-0.003	-0.026	-0.009	-0.000	-0.011	0.001	-0.021	0.084
	128.0	0.004	0.003	0.005	0.006	0.000	-0.020	0.018	0.001	0.013	0.016
	256.0	0.029	0.015	-0.002	0.020	0.008	0.021	-0.007	-0.002	0.008	-0.100
Dimension of the latent space	2.0	-0.042	-0.017	0.004	-0.054	-0.030	0.138	0.012	0.033	0.025	-0.010
	8.0	0.021	0.022	0.004	0.006	0.006	0.015	0.019	-0.009	0.013	0.025
	10.0	0.026	0.013	-0.003	0.016	0.004	0.008	0.021	-0.021	0.023	-0.011
	16.0	0.005	0.004	-0.002	0.007	0.001	-0.016	0.013	-0.010	0.003	-0.003
	32.0	-0.002	-0.005	-0.002	0.012	0.009	-0.042	0.002	-0.001	0.002	-0.019
	50.0	0.004	-0.003	0.008	0.000	0.001	-0.028	-0.016	-0.004	-0.014	0.005
	64.0	-0.017	-0.004	0.005	0.004	0.004	-0.047	-0.026	0.010	-0.009	0.000
	128.0	0.004	-0.009	-0.013	0.009	0.005	-0.026	-0.025	0.002	-0.043	0.014
Layers	1.0	-0.021	0.019	-0.015	-0.008	-0.008	-0.040	-0.018	0.000	0.001	-0.081
	2.0	0.021	-0.019	0.015	0.008	0.008	0.040	0.018	-0.000	-0.001	0.081
Reconstruction loss	nb	-0.003	0.004	-0.004	-0.004	-0.002	0.002	0.013	0.007	0.007	0.011
	zinb	0.003	-0.004	0.004	0.004	0.002	-0.002	-0.013	-0.007	-0.007	-0.011

Figure S14: Heatmap visualization of the mean effect of each parameter value of scVI on its AMi. Each columns corresponds to a dataset. The rows are split by parameter and their values, the numbers show the average effect of that parameter value on the AMI compared to the mean AMI for scVI on that dataset.
These effects come from a factorial ANOVA.

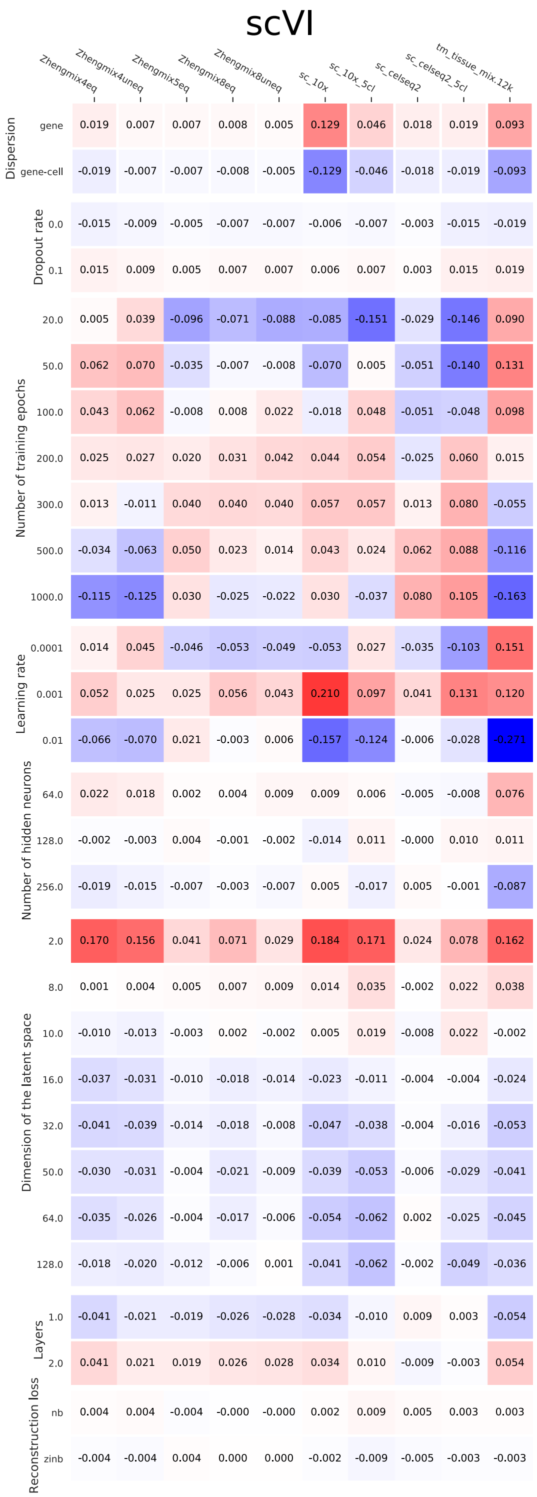

Figure S15: Heatmap visualization of the mean effect of each parameter value of scVI on its silhouette. Each columns corresponds to a dataset. The rows are split by parameter and their values, the numbers show the average effect of that parameter value on the silhouette compared to the mean silhouette for scVI on that dataset.
These effects come from a factorial ANOVA.

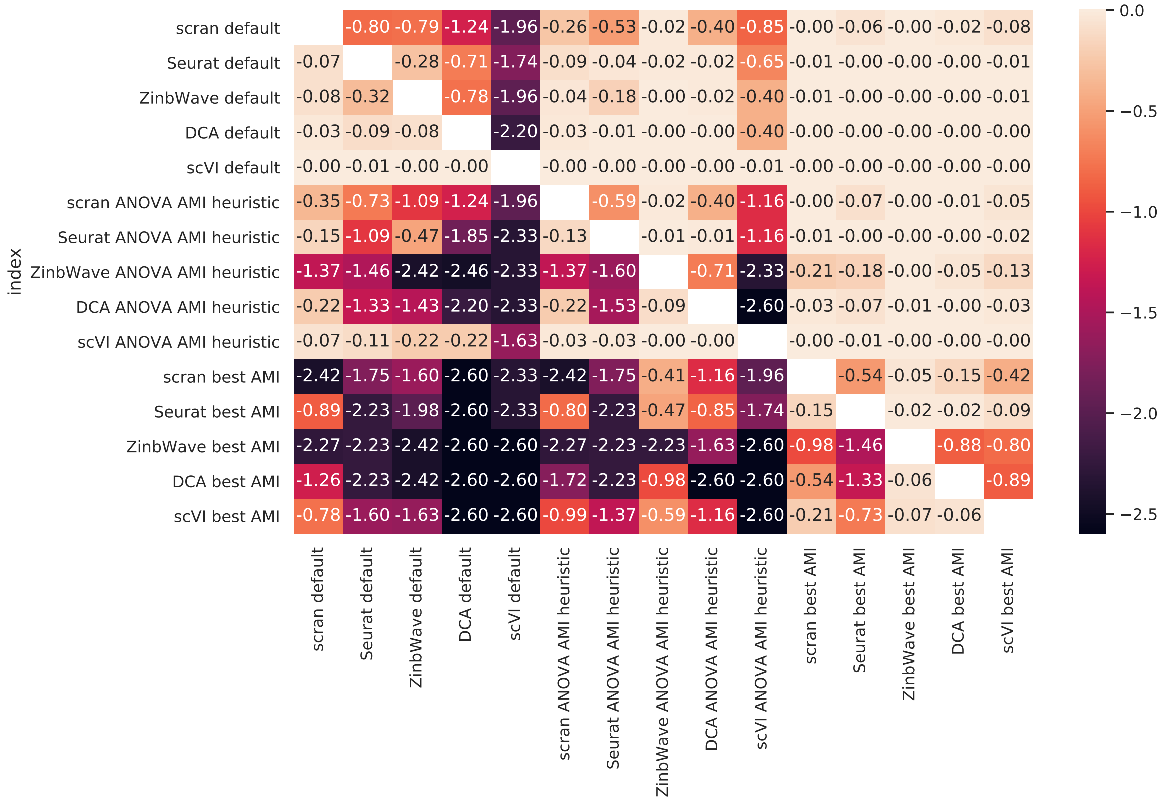

Figure S16: log base 10 p-values for the wilcoxon one-way test between the various methods and parameter configurations in AMI. A p-value of 0.05 coresponds to -1.3 in log base 10. The test is used to see if the method and parameters in the row achieve a higher AMI than the one in the column.

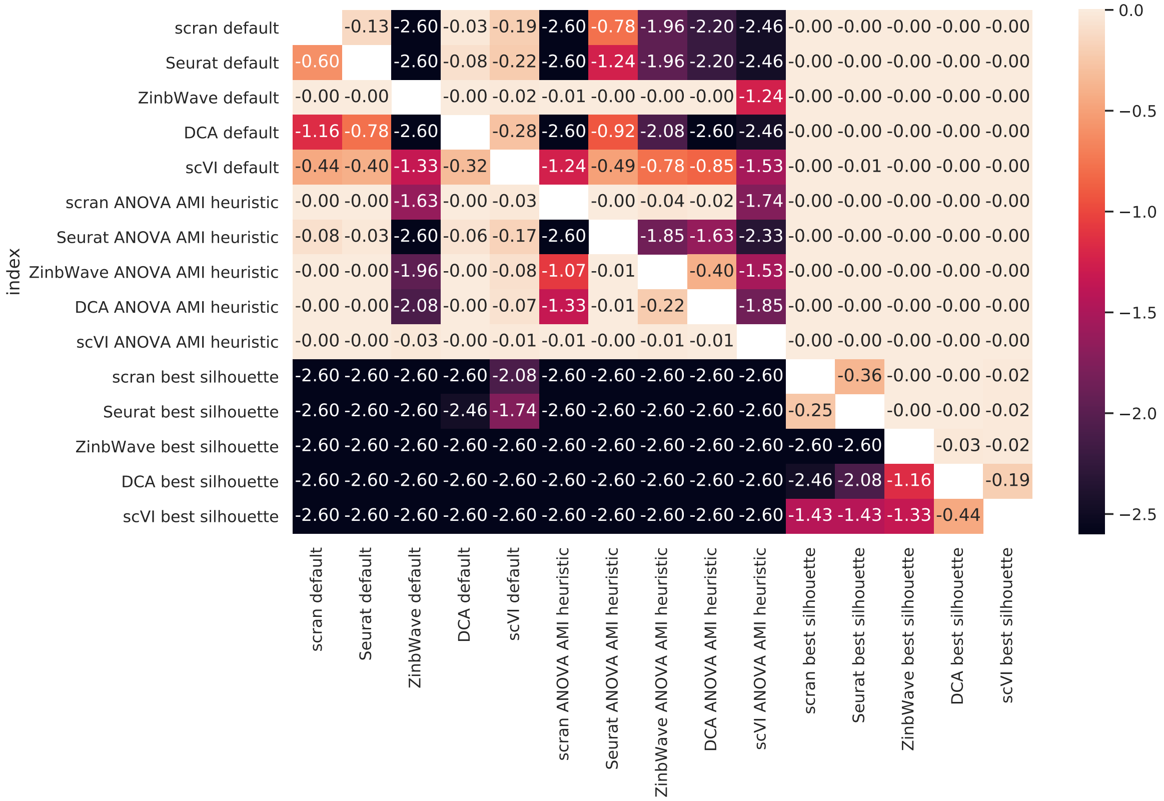

Figure S17: log base 10 p-values for the wilcoxon one-way test between the various methods and parameter configurations in silhouette. A p-value of 0.05 coresponds to -1.3 in log base 10. The test is used to see if the method and parameters in the row achieve a higher silhouette than the one in the column.
