# Supplementary material for: Tuning parameters of dimensionality reduction methods for single-cell RNA-seq analysis
Source: Genome Biol. 2020 Aug 24;21:212. doi: 10.1186/s13059-020-02128-7 (PMC7444048; doi:10.1186/s13059-020-02128-7)
Supplement: Supplementary file 2 — Additional file 2 Review history. [file 13059_2020_2128_MOESM2_ESM.docx]

Review History

**First round of review**

**Reviewer 1**

**Were you able to assess all statistics in the manuscript, including the appropriateness of statistical tests used?**

Yes, and I have assessed the statistics in my report.

**Comments to author:**

In this paper, Raimundo et al investigate how parameter tuning influences the performances of dimension reduction in the most commonly used single cell RNA-seq pipelines. I think this paper is extremely well laid out and presented. Moreover, the presented research, which is extensive and thorough, is thoughtfully discussed and put into context. In my opinion, this research points to areas in single cell analysis that urgently need improvement and hence is of great importance to the single cell community.

I have only a few comments that will mainly improve the clarity of the manuscript:

1. The authors should justify why they decided for the silhouette and AMI to investigate performance, as they are many other conceivable choices.

2. When using AMI could k-means clustering step be replaced with more commonly used clustering algorithm, such as Louvain? This would better reflect the state of the art of many single cell pipelines.

3. I feel Figure 3A is very busy. If possible it would be great if the information could be split across multiple panels.

4. For Table 3, it would be useful to quantify the effect of each parameter on the outcome.

Thank you for making your code public.

**Reviewer 2**

**Were you able to assess all statistics in the manuscript, including the appropriateness of statistical tests used?**

Yes, and I have assessed the statistics in my report.

**Comments to author:**

In this manuscript, Raimundo and colleagues evaluate the performance of 5 dimension reduction methods on 10 single-cell RNA-seq data set, in terms of the ability to distinguish between known cell types in the low-dimensional representation. Particular focus is put the impact of the parameters of the respective methods. This is a nicely written paper with well-supported conclusions. I have only a few relatively minor comments, mostly related to clarity and reproducibility.

1. The authors conclude that their heuristic way of determining the best parameters without prior knowledge of the cell types (silhouette heuristic) does not necessarily work well, and encourage more research on this topic. At the same time, they note that re-running all their experiments would take years. Thus, I think it would be very valuable if the processed data (i.e., the low-dimensional representations obtained with different parameter settings) would be made available to the community, to stimulate further research.

2. Please add some details on how UMAP was run.

3. It could be clarified in the text (p.8, l.~50) that the ANOVA AMI heuristic parameters are the same across data sets - this is made explicit only a bit below.

4. For reproducibility, please indicate the precise version of the R packages used.

5. There are a few typos and repeated words (e.g. "Euclidean Euclidean"), particularly in the Methods section.

6. Fig. 3A is a nice summary of the results. Perhaps the colors of the "default" and "best" parameters could be a bit more distinct, I found it hard to compare the performances of the default parameters across methods.

**Authors Response**

**Point-by-point responses to the reviewers’ comments:**

*We thank the reviewers for their detailed and helpful comments. We revised the manuscript accordingly, taking into account all comments. In particular, we re-ran the full benchmark to validate that the conclusions of our study do not depend on the choice of the clustering algorithm used (we tested Louvain and hierarchical clustering, in addition to k-means), or on the metrics used to assess the quality of clustering (we tested ARI, in addition to silhouette and AMI). We also reformatted some figures to increase readability, and provide as supplementary data a large collection of pre-computed single cell embeddings to stimulate further research on heuristics to choose optimal parameters.*

*All modifications are highlighted in red in the revised manuscript, and we provide below point-by-point answers to the reviewers’ questions. Comments from reviewers are indicated in bold characters, and our responses in normal characters.*

*We remain available for further clarifications if needed, and look forward to hearing from you.*

*Best regards, The authors.*

Reviewer #1: In this paper, Raimundo et al investigate how parameter tuning influences the performances of dimension reduction in the most commonly used single cell RNA-seq pipelines. I think this paper is extremely well laid out and presented. Moreover, the presented research, which is extensive and thorough, is thoughtfully discussed and put into context. In my opinion, this research points to areas in single cell analysis that urgently need improvement and hence is of great importance to the single cell community.

I have only a few comments that will mainly improve the clarity of the manuscript:

1.	The authors should justify why they decided for the silhouette and AMI to investigate performance, as they are many other conceivable choices.

*We agree with the reader that there exist many conceivable choices to assess if an embedding of cells maps cells of the same class near each other. However, many measures are correlated to each other, so we decided to report AMI and silhouette because:*

*-	They capture two slightly different properties of the distribution of cells. As explained in Section 2.1, “AMI directly measures how well a particular clustering algorithm recovers known cell types, and is therefore a good proxy for the performance of cell type identification as a downstream task of DR. Silhouette is a measure agnostic to any particular clustering algorithm, and measures how close a cell is to other cells of the same type compared to cells of different types; for the silhouette to be large, cell types must not only be separated, but also form compact clusters far from each other.”*

*-	They are standard measures often used in that context, eg, [10] reports NMI, a scaled version of AMI, and adjusted rand index (ARI); [11] report ARI; [18] reports silhouette; [20] reports silhouette, ARI and NMI; [23] report AMI, ARI and homogeneity.*

*We note that, in addition to silhouette and AMI/NMI that we report, ARI is often used in benchmarks of cell embedding methods. To check if assessing the embedding performance using ARI instead of AMI would change our conclusions, we therefore computed the ARI performance for all experiments. As shown on the plots below (which we added as Supplementary Figure 1), AMI and ARI are extremely correlated (each plot is an experiment, the x and y axes are respectively the ARI and AMI performance of k-means). We checked that using ARI instead of AMI barely changes the results reported in the paper, and does not impact the conclusions. We therefore decided to keep AMI and silhouette as main measures, and added the following sentence in Section 2.1:*

*“ Both measures are frequently used to assess the performance of cell embedding techniques [18,11,20,23,10]. Another standard measure to assess the ability of a clustering algorithm to recover known classes is the adjusted rand index (AR!) [11,20,23,10]; however, we found that AM! and AR! are extremely correlated (Supplementary Figure 1) and lead to similar conclusions, so we just report results based on AM! below. ”*

2.	When using AMI could k-means clustering step be replaced with more commonly used clustering algorithm, such as Louvain? This would better reflect the state of the art of many single cell pipelines.

*This is a fair point. An advantage of k-means is that we can easily constrain the number of clusters to be equal to the true number of classes, removing one source of errors. To better reflect the state of the art of many single cell pipelines, we also tested two alternative clustering techniques to our benchmark: Louvain, as suggested by the reviewer, and hierarchical clustering (Ward’s method), another commonly used method. As shown in the plot below (new Supplementary Figure S4), the results with hierarchical clustering are basically the same as for k-means, while the performance decreases with Louvain, possibly due to using scanpy’s default parameters since we did not find a systematic way to automatically tune the resolution nor the parameters of the nearest neighbours graph on each experiment to create the desired number of clusters. We added the following paragraph to Section 2.3 to summarize these results: “ To check how the AM! results are influenced by the choice of the clustering algorithm (here k-means), we also computed the AM! performance when cells are clustered by Ward's hierarchical clustering (HC) or the Louvain algorithm [24]. As shown on Supplementary Figure S4, the performance barely changes when k-means is replaced by HC. When Louvain clustering is used, the overall performance decreases on most datasets, possibly because we used default parameters for this algorithm, while k-means and HC are run knowing in advance the correct number of clusters. The relative ordering of the different method is overall conserved, suggesting our conclusions based on AM! are robust to the clustering method.. ” We also added in section 4.2 a description of how each clustering algorithm was run.*

3.	I feel Figure 3A is very busy. If possible it would be great if the information could be split across multiple panels.

*We agree with the reviewer that Figure 3A was very busy, particularly because we superimposed the results of 4 variants (default parameters, best parameters, AMI ANOVA heuristic, silhouette heuristic). To increase readability, we modified Figure 3A to keep only 2 variants (default and best parameters), which are discussed in Sections 2.2 and 2.3. We moved the results of the AMI ANOVA and silhouette heuristics, discussed in Sections 2.4 and 2.5, to supplementary Figure S5 (while keeping a summary of these results in the main Table 2).*

4.	For Table 3, it would be useful to quantify the effect of each parameter on the outcome.

*Table 3 presents the parameter sweep (and the default values) performed for each method, and is referred to in Section 2.3, comparing the performance of the default value (studied in Section 2.2) and the best value over the grid. Quantifying the effect of each parameter on the outcome is the subject of Section 2.4, and the detailed effects are shown in Tables S1-S2 (as mentioned in the text, section 2.4).*

Reviewer #2:

In this manuscript, Raimundo and colleagues evaluate the performance of 5 dimension reduction methods on 10 single-cell RNA-seq data set, in terms of the ability to distinguish between known cell types in the low-dimensional representation. Particular focus is put the impact of the parameters of the respective methods. This is a nicely written paper with well-supported conclusions. I have only a few relatively minor comments, mostly related to clarity and reproducibility.

1.	The authors conclude that their heuristic way of determining the best parameters without prior knowledge of the cell types (silhouette heuristic) does not necessarily work well, and encourage more research on this topic. At the same time, they note that re-running all their experiments would take years. Thus, I think it would be very valuable if the processed data (i.e., the low-dimensional representations obtained with different parameter settings) would be made available to the community, to stimulate further research.

*We thank the reviewer for this very good suggestion, and are happy to make publicly available the low-dimensional embeddings we computed. While we understand that all the embeddings would be useful for the community, we are however limited by the large volume of data to store (we tested almost 1.5 million configurations, and each embedding corresponds to a file of ~2Mb, so the full dataset would weight ~3Tb). In order to help the community we decided to compute and share a medium size set of 5,000 embeddings (100 per dataset and per method) which we hope will help trigger new research. We added the following sentence to Section 2.5:*

*“ !n order to stimulate further research in that direction, we provide as supplementary data an original dataset consisting of 5,000 embeddings (100 for each single cell dataset and for each method, corresponding to 100 random choices of different parameters, including the default ones and the ones achieving the best AM! and silhouette). ”*

2.	Please add some details on how UMAP was run.

*We added the following information in section 4.6: “ The UMAP plots were generated using scanpy's (version 1.3.8) implementation with default parameters, run on all the dimensions of the low dimension representation. ”*

3.	It could be clarified in the text (p.8, l.~50) that the ANOVA AMI heuristic parameters are the same across data sets - this is made explicit only a bit below.

*Thanks for the suggestion, the reader may indeed be confused if we do not state it explicitly, so*

*we added the following sentence in Section 2.4, just after defining the ANOVA AMI heuristic:*

*“ Note that, by construction, the ANOVA AMI heuristic parameters are the same for all datasets. ”*

4.	For reproducibility, please indicate the precise version of the R packages used.

*Thanks for the suggestion, we added all software versions in the Methods section.*

5.	There are a few typos and repeated words (e.g. "Euclidean Euclidean"), particularly in the Methods section.

*Thank you for the comment. We corrected this and other typos.*

6.	Fig. 3A is a nice summary of the results. Perhaps the colors of the "default" and "best" parameters could be a bit more distinct, I found it hard to compare the performances of the default parameters across methods.

*Thank you for the advice. As explained to Reviewer 1 (question 3), we decided to move that figure in Supplementary figure S5, and keep only the performance for the default and best parameters in Figure 3.A. This should make Figure 3.A more readable while keeping all the information accessible for the reader.*

**Second round of review**

**Reviewer 1**

All my comments were addressed. This is a really nice paper!
